# New Technique to Repair Keyhole of 2195 Al-Li Alloy Friction Stir Welding Joints

**DOI:** 10.3390/ma17143418

**Published:** 2024-07-11

**Authors:** Xiangchen Meng, Xi Chen, Zhulin Han, Jingyu Yuan, Yuming Xie, Jihong Dong, Peiyun Xia, Yongxian Huang

**Affiliations:** 1 State Key Laboratory of Precision Welding & Joining of Materials and Structures, Harbin Institute of Technology, Harbin 150001, China; mengxch@hit.edu.cn (X.M.); hitchenxi2003@163.com (X.C.); hit__hanzhulin2003@163.com (Z.H.); hityuanjy@126.com (J.Y.); ymxie@hit.edu.cn (Y.X.); 2 Zhengzhou Research Institute, Harbin Institute of Technology, Zhengzhou 450046, China; 3 AVIC Manufacturing Technology Institute, Beijing 100024, China; 4 Shanghai Aerospace Equipment Manufacturer Co., Ltd., Shanghai 200245, China; xpypei@126.com

**Keywords:** 2195 Al-Li alloy, keyhole, friction stir processing, tungsten inert gas welding, mechanical properties

## Abstract

Aiming at the repairing of keyhole defects after friction stir welding of complex structures, a new method combined with tungsten inert gas welding (TIG) and friction stir processing (FSP) was proposed. The results showed that the pre-filling wire of TIG can completely fill the volumetric keyhole. FSP can refine the coarse grain area into equiaxial grains due to dynamic recrystallization, while some pore defects are eliminated. The interface bonding quality is high. The microhardness of the repairing zone with FSP is significantly stronger than that of the untreated parts. Compared to direct TIG repairing, the introduction of FSP transformed the fracture from brittle fracture to ductile fracture, and the tensile strength of the joint was increased by 131.7%, realizing the high-quality repairing of keyhole defects in 2195 Al-Li alloy.

## 1. Introduction

Al-Li alloys exhibit significant potential for aerospace applications owing to their exceptional specific strength and low density, which have been partially employed in rocket fuel storage tanks as well as aircraft bearing components [1,2,3,4]. They have an improved capability to withstand localized deformation [5]. The 2195 Al-Li alloy exhibits ultra-high strength, excellent malleability, and low-temperature performance and has been extensively utilized in the fabrication of cryogenic propellant tanks for spacecraft launch vehicles [6,7]. The 2195 Al-Li alloy exhibits certain welding defects, such as porosity and thermal cracking, via the conventional fusion welding method. Li has a high melting loss rate, leading to low joint strength compared to that of the base metal [8]. Therefore, it is crucial to develop efficient welding and repair techniques for attaining reliable joints of Al-Li alloys.

Friction stir welding (FSW), as an innovative solid-phase joining technology, presents numerous advantages over conventional fusion welding methods, encompassing low heat input, high joint quality, and small welding distortion [9,10]. It has been extensively investigated and applied in the welding of lightweight alloys [11,12]. When employed for Al-Li alloy welding, the material remains solid due to the low welding temperature, which effectively mitigates defects arising from the loss of Li elements during melting. FSW in an Al-Li alloy limits the occurrence of defects while achieving a high strength coefficient [13,14]. In the welding-tool lifting stage, the pin is removed from the base material. The keyhole defect occurs at the end of the FSW weld, which diminishes the effective bearing area of the weld and compromises its load-bearing capacity. It is necessary to solve the problem of repairing keyhole defects in order to realize the full FSW of the launch vehicle tank and other components.

Building upon FSW principles, friction plug welding (FPW), active–passive filling friction stir repairing (A-PFFSR), and filling friction stir welding (FFSW) were proposed [15]. FPW relies on high-speed rotation of the plug to generate substantial heat input that transforms the material and tip into a thermoplastic state; the interface was eliminated by applying axial feed [16,17,18]. For products with a complex structure, large volume, and large support tools that cannot be set on the back of the welded part, friction pull plug welding (FPPW) technology enables the effective repair of FSW keyholes [19]. Ji et al. [11,20,21] devised A-PFFSR, which consists of two stages: active repairing and passive repairing. The active repairing process employs a series of non-rotating welded tools with increasing diameters to progressively fill metallic material from the side wall to the bottom of the hole in the keyhole. However, in the passive repairing process, materials with equal diameter but greater height are used initially to fill up the keyhole before employing larger diameter welded tools for further repairs. Huang et al. [22,23] employed FSW with a stirring pin composed of the same material as the keyhole. Both the stirring pin and the keyhole undergo simultaneous thermoplastic flow throughout the weld-filling process. Ultimately, upon withdrawal of the welding tool, only the conical portion of the stirring pin remains within the keyhole, thereby achieving effective weld filling. FFSW can restore quasi-equal strength of the welded keyholes.

The aforementioned methods effectively mitigate keyhole defects and enhance the mechanical properties of joints when employing the appropriate welding parameters. In order to realize the high-quality repairing of keyhole defects in 2195 Al-Li alloy, to eliminate severe joint softening, and to avoid the requirement of the back-supported body in solid-phase joining, TIG combined with a friction stir processing (FSP) technique was proposed via the TIG filling and FSP microstructural modification, which has the characteristics of simple welding process equipment, convenient operation, solid-phase repairing, and high performance. This method improved the joint structure, eliminated the defects in the fusion welding process, and improved the mechanical properties of the components. Therefore, this paper focuses on surface morphology, microstructural characteristics, and mechanical properties. This approach provides a theoretical foundation for FSW keyhole-repairing technology and efficient joining of the 2195 Al-Li alloy. 

## 2. Experimental Procedures

In this study, a sheet of 2195 Al-Li alloy with a thickness of 6 mm was used as the base metal along with TIG welding wire. The TIG filling material was the narrow strip cut from the 2195 Al-Li alloy sheet via wire-cut electrical discharge machining, which was further polished before the TIG filling process. The chemical composition of 2195 Al-Li alloys is shown in Table 1.

**Figure 1:**
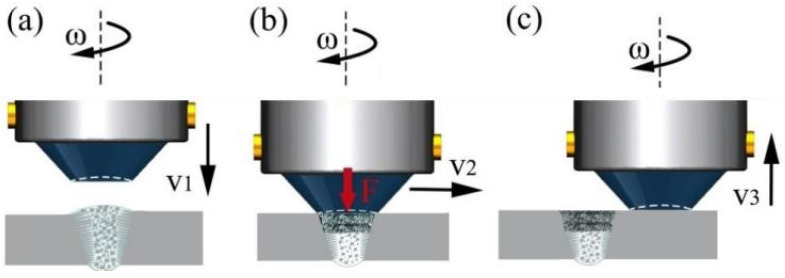
Schemes of the friction stir processes: (**a**) the welding tool is lowered and applied to the repair joint, (**b**) the welding tool is moved for FSP, and (**c**) the welding tool is lifted after traveling a certain distance.

The repair of the keyhole involved a combination of FSP following fusion welding. Initially, manual TIG welding was employed to fill the keyhole in the FSW joint of 2195 Al-Li alloy. An arc was used for preheating the keyhole prior to the welding process. The fill wire for TIG welding had a fixed current of 140 A and remaining height control of 2 mm. Following TIG welding, the FSP process was implemented to refine coarse grains resulting from high heat input during TIG welding and enhance the mechanical properties of the joint. The FSP-repair process and parameters were as follows. The FSP used a gantry CNC friction stir welding machine (FSW-3LM-003, Wanzhou Welding Co., Ltd., Harbin, China). A steel pinless welding tool with a shoulder diameter of 20 mm was utilized. The area filled by TIG was treated with a tool rotation speed of 600 r/min and a plunge rate of 1 mm/min. After plunging into the plate surface by 0.1 mm, it remained stationary for 2 s before moving at a welding speed of 200 mm/min over a distance of 20 mm. The repair process is visually depicted in Figure 1. 

**Figure 2:**
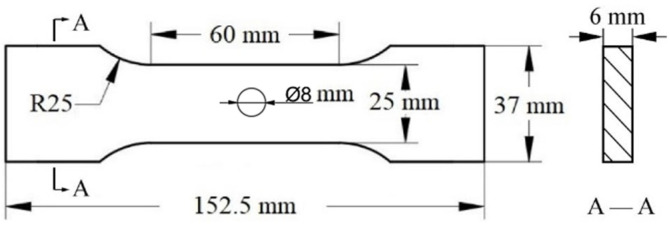
Schematic diagram of tensile specimens.

The surface morphology and microstructure analysis of the joints in the cut and polished specimens were characterized by using an optical microscope (OM, Keyence VHX-1000E, Osaka, Japan). The grain size was evaluated via the lineal intercept method. Subsequently, the fracture morphology was examined on a Zeiss-Supra55 field emission scanning electron microscope (SEM, Oberkochen, Germany) after metallographic polishing and corrosion. Energy dispersive spectroscopy (EDS) of SEM was adopted to analyze the precipitated phases and confirm the kinds of precipitation phases in various regions of the metallographic specimen. Tensile testing was performed on a universal material testing machine (AG-IC20KN, Shimadzu, Kyoto, Japan). The tensile specimens were extracted via wire-EDM. Each method corresponds to three tensile specimens, and the tension speed was 3 mm/min. Figure 2 demonstrates the tensile test method and specimen dimensions following the GB/T2651-2008 standard [24] for analyzing the mechanical properties of the joints. These samples were manually polished to 400 grits to eliminate the surface condition caused by the EDM process and avoid any potential notch-like surface features. The cross-sectional area of the sample perpendicular was measured with vernier calipers to determine the engineering stress value. After tensile deformation at room temperature, the fracture morphologies were observed via Zeiss-Supra55 scanning electron microscope (SEM). 

**Figure 3:**
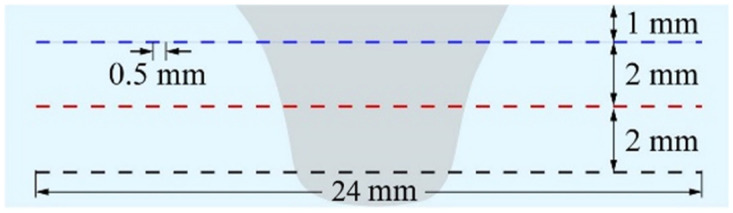
Distribution of hardness test points.

The microhardness of various areas within the repair joint cross-section was tested on an HXD-1000TM Vickers microhardness tester (Shanghai optical instrument factory no.1, Shanghai, China) at every 0.5 mm interval under a constant load of 200 g applied for 10 s. Three lines were taken into the test along the thickness direction. The spacing between the lines is 2 mm, as shown in Figure 3. 

## 3. Results

### 3.1. Repairing Formation and Microstructure Characterization

**Figure 4:**
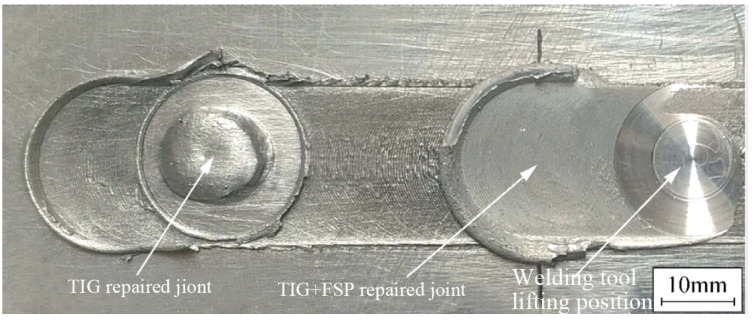
Surface morphologies of single TIG and TIG combined with FSP-repaired joints.

Manual Tungsten Inert Gas (TIG) welding was employed to address the keyhole in Al-Li alloy FSW joints, which was subsequently followed by FSP for grain structure refinement and mitigation of adverse effects resulting from TIG repairing. Figure 4 illustrates the surface morphology of single TIG and TIG combined with FSP-repaired joints. It can be observed that the TIG-repaired joint exhibits a well-formed appearance, where the keyhole has been sufficiently filled under the condition without backing rigid support. Following the FSP, effective utilization of a large shoulder on a pinless welding tool facilitates the leveling of residual height resulting from single TIG repair, thereby yielding a refined and smooth joint surface. 

**Figure 5:**
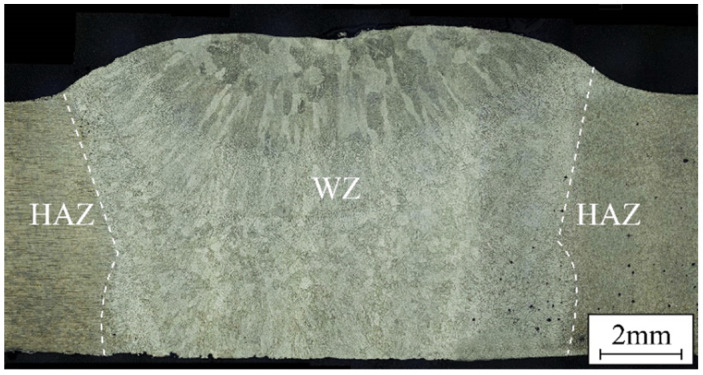
Macrostructure of single TIG-repaired joint.

**Figure 6:**
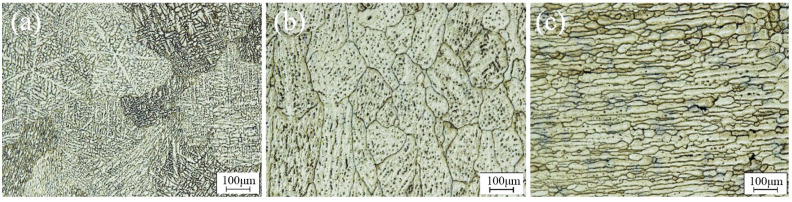
Microstructure of TIG-repaired joint in various zones: (**a**) upper WZ, (**b**) lower WZ, and (**c**) HAZ.

Figure 5 and Figure 6 show the macrostructures and microstructures of the single TIG-repaired joint via an optical microscope. The keyhole was fully filled and closely bonded to the base material. The joints could be divided mainly into the weld zone (WZ) and the heat-affected zone (HAZ). Since the keyhole was preheated with an arc before TIG filling, the joints are tightly bonded. Due to the high heat input of TIG repairing, the filled joint exhibits a typical casting structure. The plasma airflow exhibited significant cooling close to the joint surface, facilitating efficient heat dissipation and promoting the complete development of columnar grains on the joint surface. The side surface of the keyhole at the bottom of the joint was partially melted, resulting in coarse equiaxed grain morphology due to the same heat dissipation gradient.

Figure 6a,b show the microstructure of the upper and lower layers of the WZ, respectively. The upper layer of the WZ consisted of columnar crystals and coarse equiaxed grains; the crystallite size can reach 425.13 ± 19.1 μm. This observation suggests that an imbalanced crystallization process leads to dendrite segregation, and the low melting eutectic structure between dendrites increases the tendency to produce thermal cracks. The heat input in the lower layer of the WZ was relatively small, reducing grain coarsening degree (169.66 ± 17.1 μm) and segregation. The heat conducted by the WZ affected solely the HAZ without inducing melting or recrystallization, thereby preserving the rolled grain structure with a significant grain size, as depicted in Figure 6c. The high heat input in TIG repair inevitably causes grain coarsening, this effect also ensures that the keyhole is bonded tightly to the filler material. Therefore, understanding how to solve the grain coarsening and improve mechanical properties is important. 

**Figure 7:**
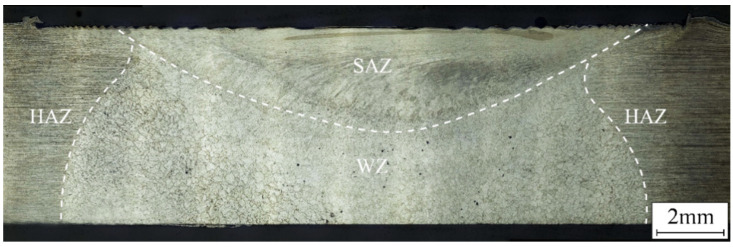
Macroscopic morphology of TIG combined with FSP-repaired joint.

Figure 7 shows the macroscopic morphology of the joint with FSP on a TIG-repaired joint via OM. The top surface of the FSP-repaired joint became flatter and smoother. The joint could be divided into the shoulder-affected zone (SAZ), the WZ, and the HAZ. In the FSP, the top surface of the workpiece was subjected to the stirring effect of the concave shoulder, and the plasticized materials experienced stirring and forging actions. The excess height formed during the TIG repair process was completely compacted in the joint, resulting in a smooth joint surface and reducing stress concentration. The weld zone was characterized by equiaxed grains, and the HAZ retained the rolling structure.

**Figure 8:**
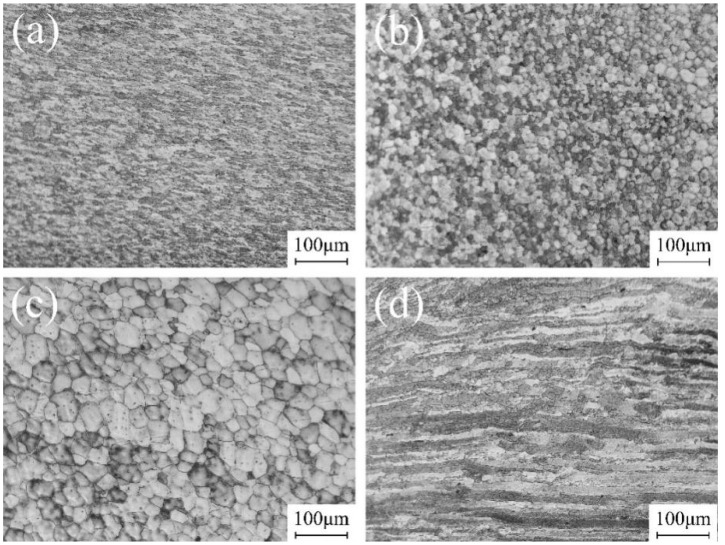
Microstructure of TIG combined with FSP-repaired joints: (**a**) SAZ, (**b**) junction of SAZ and WZ, (**c**) WZ, and (**d**) HAZ.

The microstructure of the joint treated with FSP and repaired by TIG is illustrated in Figure 8. Figure 8a displays the microstructure within the SAZ, where significant FSP action takes place under the thermo-mechanical effect, plastic deformation and dynamic recrystallization, resulting in a transformation from coarse columnar and equiaxed grains to dynamically recrystallized grains, exhibiting a curved and elongated structure. Fine grains make it possible for plastic deformation to occur more evenly under external force (26.31 ± 0.64 μm), decreasing the tendency for cracks to expand. These grains also exhibited elongated morphology under mechanical stirring, leading to the dislocation density increase greatly, increasing the strength and hardness. The influence of FSP diminishes, resulting in larger recrystallized grains with limited tensile effects as the distance from the shoulder increases. At the junction between the SAZ and the WZ, the subgrain boundaries continue to absorb dislocations under the action of thermal cycling. The orientation difference of the grain boundaries changes, finally forming the equiaxed crystals (19.87 ± 0.92 μm) due to reduced mechanical action from the shoulder, resulting in an equiaxed grain morphology as depicted in Figure 8b. The WZ region corresponds to the original TIG weld nugget zone, which experienced minimal impact from FSP treatment. In this region, the recrystallization process does not occur, and coarse grains (33.63 ± 1.10 μm) of fusion welding are retained, as shown in Figure 8c, becoming a weak part in the joint. Figure 8d illustrates the microstructure within the HAZ. Due to secondary heat input from both TIG and FSP processes, the HAZ retains its rolled morphology similar to that of parent material but undergoes severe grain coarsening. The average crystallite size was 139.41 μm.

### 3.2. Precipitate Characterization

**Figure 9:**
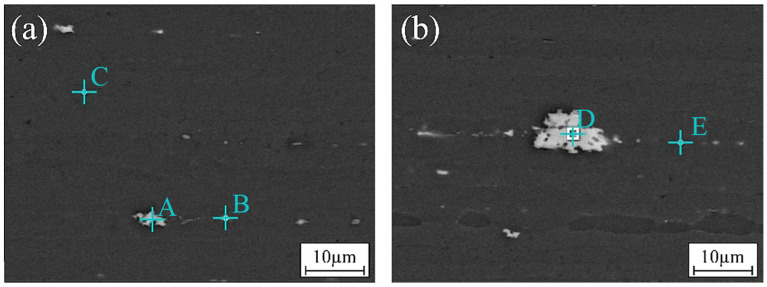
Precipitation distribution and analysis of base material: (**a**) typical area and (**b**) special area.

The alloying elements of 2195 Al-Li alloy are mainly Cu, about 4%wt. All of the precipitations contain Al and Cu elements. Al-Li alloys derive their highest strength from the combination of cold work and annealing for homogenous dispersion of the T_1_ precipitate [25,26]. In order to understand the strengthening effect of precipitate phases in welding, EDS was adopted to analyze the precipitate phases in the welding joints in terms of their types, shapes, and sizes. Figure 9a shows the distribution morphology of precipitations in the base material. Figure 9b shows the large precipitations found in the base material. Due to the properties of the Li element, it is difficult to visualize it on the spectrum. As a result, the Li element was not included in the spectrum test. The alloy element content at each point in Figure 9 is shown in Table 2. Points A and D show the irregular and large precipitate phases in the base material. The phases contain Al, Cu, and Zr elements. Small rod-shaped precipitate phases were found on grain boundaries, as shown in Point B. It was presumed to be the Al-Cu phase. Points C and E show the finer and nearly circular precipitations on grain boundaries. During the fusion welding repair process of the 2195 Al-Li alloy, the evaporation of the low-melting-point Li element in the alloy inevitably occurs, leading to a reduction in the δ’ phase and T_1_ phase, thereby diminishing their strengthening effect. Hence, judiciously reducing heat input during welding can effectively minimize burn loss of low-melting-point strengthening phases and consequently enhance joint performance. In the TIG repair process, substantial dissolution of the major strengthening phase T_1_ may take place. Based on phase diagrams and atomic percentages, it is hypothesized that typical points contain the T2 phase (Al6CuLi3) and TB phase (Al7Cu4Li).

**Figure 10:**
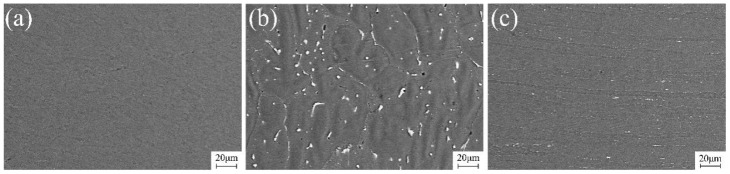
Precipitation phase morphology and distribution of TIG combined with FSP-repaired joint: (**a**) SAZ, (**b**) WZ, and (**c**) HAZ.

The morphology and distribution of the precipitation phases in FSP-repaired joints are depicted in Figure 10. In the SAZ, dynamic recrystallization occurred due to thermal–mechanical action, resulting in complete fragmentation and a solid solution of the precipitation phases generated via TIG repairing into the matrix structure. As a result, there are almost no observable precipitation phases, and the microstructure becomes homogeneous. In the WZ, larger grains are observed due to high heat input during TIG repair; the distribution of precipitates at the grain boundaries was remarkably dense. The presence of grain boundaries and dislocations could reduce the activation energy for precipitate formation, promoting the development of precipitates. This results in the precipitated phase preferentially nucleating at the grain boundary and growing and coarsening rapidly. The coarse precipitation affects the grain boundary stability and grain boundary strength, reducing the plasticity of the material and leading to an adverse effect on the property of the joint. The thermal influence in the HAZ preserves the rolled-state morphology of the microstructure with partially elongated precipitates observed along grain boundaries. The amount of precipitation near the weld center decreased significantly, indicating that part of the precipitation dissolved in the matrix under the action of thermal cycling. The property of the material is improved under the action of solid-solution strengthening. The rolling state is still maintained in the distance, and grain coarsens under the influence of heat. The morphology and distribution of these precipitation phases significantly impact joint performance.

Compared with single TIG repair, TIG combined with FSP can improve the repairing formation and render the joint smooth and flat, reducing stress concentration. The grain of the joint is refined from the coarse columnar grains to the fine equiaxed grains through mechanical action. The movement and expansion of dislocations are hindered by grain boundaries. The increase in grain boundaries further enhances the hindrance effect, leading to an increase in material strength. In this process, the precipitation of segregation caused by TIG repair will also be broken and redistributed in the WZ with the thermo-mechanical action of FSP, achieving the homogenization of the tissue. As a result, the joint strength of TIG combined with FSP is greater than that of TIG repair; strength and hardness are improved.

### 3.3. Mechanical Properties

#### 3.3.1. Hardness Distribution

**Figure 11:**
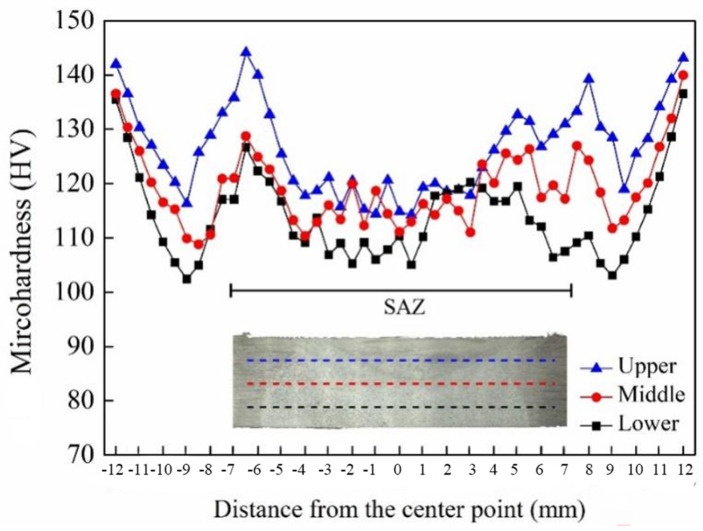
Microhardness distribution of the TIG combined with FSP joints.

The microhardness distribution of a joint repaired by using TIG followed by FSP is presented in Figure 11. It can be observed that there were variations in hardness along the zones, which were associated with microstructural changes after welding and phase transformations. In terms of thickness direction, it can be observed that the overall hardness of the top layer is higher than that of both the middle and bottom layers. The top microhardness points are located in the SAZ, which undergoes dynamic recrystallization under the mechanical action of FSP. In this area, the grains are the smallest and have a more uniform structure. The composition, morphology, and distribution of the precipitated phase have a significant influence on the hardness [27]. The precipitated phase was distributed uniformly in the matrix. Under the multiple action of solid-solution strengthening and fine-crystal strengthening, resulting in a hardness ranging between 118 ± 2.5 HV. The bottom layer is situated in the TIG fusion zone, which is severely affected by TIG heat input, leading to significant grain coarsening, and the second phases were precipitated seriously. The result is a relatively lower hardness of approximately 105 HV. The lower hardness of the WZ was related mainly to the fusion filling, Li element evaporation, and Cu element segregation to the grain boundaries. A large amount of precipitations appeared in the weld, as depicted in Figure 10b. The degree of dissolution and coarsening of the precipitated phase are relatively high, and the dislocation density is reduced, which causes the hardness to decrease. The microhardness of the intermediate layer lies in the transition zone between TIG repair and FSP, with finer equiaxed grains compared to those in the lower metal, resulting in an overall hardness ranging between 114 ± 4.8 HV. The hardness distribution is uneven along the transverse direction; there is a decrease in hardness within the WZ and the HAZ compared to that of the base material. However, at the edge of the SAZ and the HAZ, there is an increase in hardness, which becomes similar to that of the base material (135 ± 1.9 HV). The occurrence of this phenomenon can be ascribed to the combined effects of FSW, TIG repair, and FSP. The high hardness observed near the HAZ during the solidification process after melting is mainly due to its close proximity during the short liquid phase followed by a fast solidification time. As a result, precipitated phases can be uniformly distributed while being anchored to dislocations, which significantly increase microhardness within this area.

#### 3.3.2. Tensile Properties

**Figure 12:**
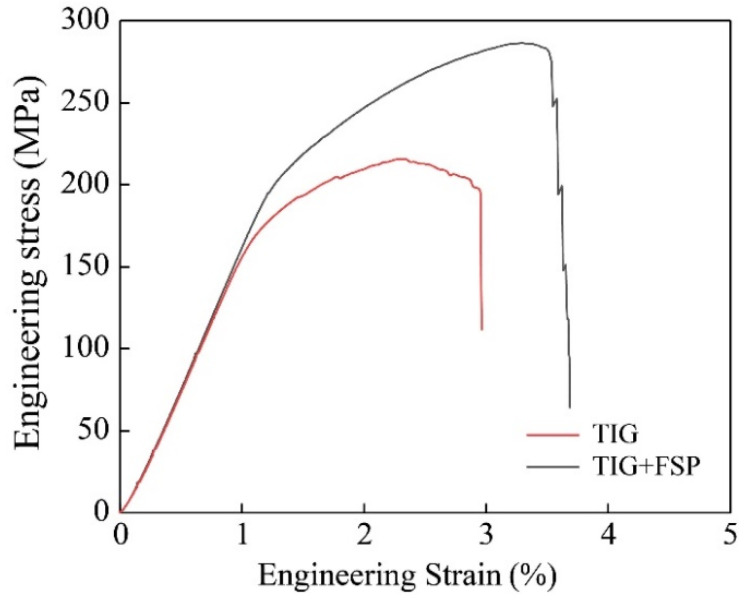
Engineering stress–strain curves of single TIG and TIG combined with FSP.

The typical engineering stress–strain curves for TIG-repaired joints and TIG combined with FSP-repaired joints are presented in Figure 12. Typical mechanical properties are summarized in Table 3. The ultimate tensile strength of the TIG-repaired joint measures 215 ± 4.5 MPa, equal to 54.3% of the weld strength. The TIG filling resulted in microstructure deterioration. The second phase precipitated along coarse grain boundaries and reduced tensile properties. Conversely, the TIG combined with the FSP-repaired joint exhibits a strength of 283 ± 5.5 MPa, equal to 74.7% of the weld strength. Incorporating FSP into TIG repair enhances the strength of the joint by 31.7% compared to single utilization of TIG repairing. FSP solved the problem of excessive heat input causing grain coarsening and the precipitation of large particle alloys in TIG filling. It refined the coarse grain area into equiaxial grains due to dynamic recrystallization; the grain size was reduced from 425 μm to 13.9 μm. According to the Hall–Petch relationship, fine grains are in favor of optimizing the internal microstructure of the joint, thereby improving the mechanical structure. The fine grain and uniform structure also increase the elongation of the joint from 3.0% to 3.7%. Meanwhile, the coarse, reinforced phase precipitated by TIG repair was dissolved in the matrix. Some pore defects were eliminated, and then the strength and toughness of the joint were improved. 

#### 3.3.3. Fracture Surface Morphologies

**Figure 13:**
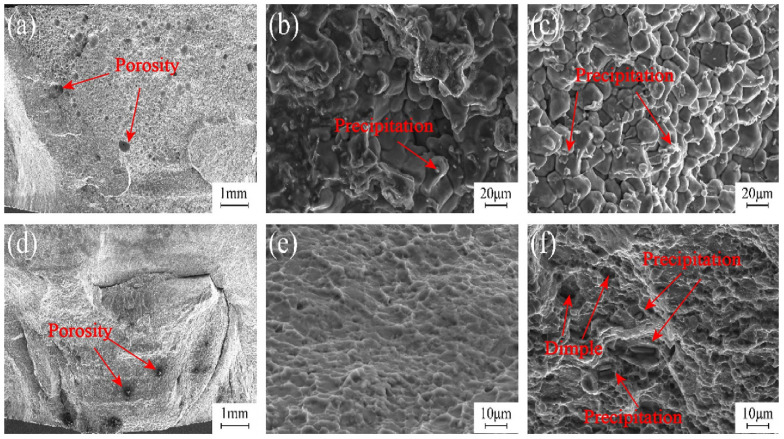
Tensile fracture surface morphologies of joints: fracture morphologies of TIG-repaired joints of (**a**) low magnification and (**b**,**c**) high magnification; (**d**–**f**) fracture surface morphologies of TIG combined with FSP of (**d**) low magnification and (**e**,**f**) high magnification.

The SEM images in Figure 13 depict the fractured surface morphologies of joints by using single TIG and TIG + FSP techniques. In Figure 13a, it was evident that the fracture occurred within the WZ during single TIG repair. Porosities from TIG filling reduce the effective bearing area, which becomes the starting point of crack initiation. The coarse precipitations of the TIG repair joint are continuously concentrated on the grain boundary, while the precipitations in the grain are relatively small. The presence of coarse precipitates at the grain boundaries weakened the bonding strength, and it was difficult to coordinate under stress, which created pathways for crack propagation, resulting in a distinct intergranular brittle fracture pattern as illustrated in Figure 13b,c. The SEM image of the joint repaired via TIG + FSP reveals the fracture location at the interface between the WZ formed via TIG repair and FSP, as depicted in Figure 13d. The hardness of the upper part of the joint is larger; the tissue is more evenly distributed. The fine structure and more sub-structure result in better mechanical properties. The coarse-grained fusion part below the joint is retained, and a large amount of precipitate phase is precipitated at the grain boundary, resulting in a lower microhardness and a weak link in the joint. This observation suggests that FSP reinforces the weld zone created through TIG repair. Figure 13e,f illustrate fracture surface morphologies characterized by numerous dimples, indicating a prominent ductile fracture. The existence of the second phase particles increases the resistance of crack propagation, which enhances the strength and toughness of the material. Bigger and deeper dimples can contain more microscopic defects, such as microscopic holes and second-phase particles, increasing the elongation rate. By correlating the analysis of fracture morphology with tensile test results, it can be inferred that FSP plays a significant role in augmenting the mechanical properties of TIG-repaired joints.

## 4. Conclusions

In order to repair the keyhole defects in FSW-repaired 2195 Al-Li alloy, a repairing method that combined TIG with FSP was proposed. Based on the investigated results, the specific conclusions can be deducted as follows:

(1) FSP, after filling via TIG welding, was proposed to repair the keyhole defects in an FFSW joint of 2195 Al-Li alloy. The joint with good surface formation was obtained via low heat input and pressure.
(2) TIG effectively achieved complete filling of the keyhole and realized the interfacial bonding of the repairing zone. FSP transformed the coarse casting microstructures in the TIG filling zone into fine and equiaxed forging microstructures and broke the precipitates at the grain boundary, which was attributed to the uniform microstructures.
(3) The tensile strength of FSP after filling via TIG reached 283 MPa, equivalent to 74.7% of the high-quality joints. Compared with the single TIG repairing, the tensile strength was improved by 31.7%. The fracture mode was changed from cleavage in TIG repairing to a mixed-fracture mode of cleavage and ductile characteristics in TIG + FSP.

## Figures and Tables

**Table 1:** The chemical composition of the base metal (wt%).

Cu	Li	Ag	Mg	Zr	Al
4.00	1.00	0.27	0.25	0.11	Bal.

**Table 2:** Alloying element content in each energy spectrum point in Figure 8 (at.%).

Location	Al	Cu	Zr
A	60.46	38.41	1.13
B	75.69	23.92	0.39
C	87.24	12.77	--
D	59.54	39.61	0.85
E	87.61	11.89	0.50

**Table 3:** Tensile properties of the joint in different states.

State	Tensile Strength/MPa	Elongation/%
Sound FSW joints	395 ± 3.1	3.5 ± 0.2
TIG	215 ± 4.5	3.0 ± 0.1
TIG + FSP	283 ± 5.5	3.7 ± 0.3

## Data Availability

The original contributions presented in the study are included in the article, further inquiries can be directed to the corresponding authors.
